# Expression Patterns of miRNA-423-5p in the Serum and Pericardial Fluid in Patients Undergoing Cardiac Surgery

**DOI:** 10.1371/journal.pone.0142904

**Published:** 2015-11-12

**Authors:** Shoichi Miyamoto, Shunsuke Usami, Yasuhide Kuwabara, Takahiro Horie, Osamu Baba, Daihiko Hakuno, Yasuhiro Nakashima, Masataka Nishiga, Masayasu Izuhara, Tetsushi Nakao, Tomohiro Nishino, Yuya Ide, Fumiko Nakazeki, Jun Wang, Koji Ueyama, Takeshi Kimura, Koh Ono

**Affiliations:** 1 Cardiovascular Center, Tazuke Kofukai Medical Research Institute, Kitano Hospital, 2-4-20 Ohgimachi, Kita-ku, Osaka, Japan; 2 Department of Cardiovascular Medicine, Graduate School of Medicine, Kyoto University, 54 Shogoin-kawahara-cho, Sakyo-ku, Kyoto, Japan; The University of Tokyo, JAPAN

## Abstract

**Background:**

Recently, it has been reported that specific microRNA (miRNA) levels are elevated in serum and can be used as biomarkers in patients with cardiovascular diseases. However, miRNAs expression profiles and their sources in pericardial fluid (PF) are unclear.

**Methods and Results:**

The purpose of this study was to identify the levels of miRNAs in PF in relation to those in the serum in patients undergoing cardiac surgery. Serum (S) and PF from patients undergoing coronary artery bypass graft (CABG) due to stable angina pectoris (sAP) and unstable AP (uAP) and aortic valve replacement due to aortic stenosis (AS) were analyzed for the detection of miRNAs. We named these samples S-sAP, S-uAP, S-AS, PF-sAP, PF-uAP, and PF-AS, respectively. We first measured the levels of miR-423-5p, which was recognized previously as a biomarker for heart failure. miR-423-5p levels were significantly higher in PF than serum. Although there was no difference in miR-423-5p levels among the PF-AS, PF-sAP, and PF-uAP, its levels were significantly elevated in S-uAP compared with those in S-AS and S-sAP. In order to clarify the source of miR-423-5p in PF, we measured the levels of muscle-enriched miR-133a and vascular-enriched miR-126 and miR-92a in the same samples. miR-133a levels were significantly higher in serum than in PF, and it was elevated in S-uAP compared with S-AS. miR-126 level was significantly increased in serum compared with PF, and the level of miR-92a the similar tendency. miR-423-5p is located in the first intron of *NSRP1*. There is another miRNA, miR-3184, encoded in the opposite direction in the same region. *In vitro* experiments indicated that the duplex of miR-423-5p and miR-3184-3p was more resistant to RNase than the duplex of miR-423-5p and miR-133-3p, which may help to stabilize miR-423-5p in the PF.

**Conclusions:**

Our results suggested that miR-423-5p is enriched in PF, and serum miR-423-5p may be associate with uAP. Its expression pattern was different to that of muscle- and vascular-enriched miRNAs, miR-133a, miR-126, and miR-92a.

## Introduction

MicroRNAs (miRNAs; miRs) are single-stranded, small, non-coding RNAs which act as post-transcriptional regulators of gene expression [1,2,3]. Each miRNA has been shown to regulate the expression of multiple genes. Conversely, the expression of each gene can be regulated by many miRNAs. Thus, well over one-third of human protein-coding genes appear to be regulated by miRNAs [4].

MiRNAs have been identified as key regulators of complex biological processes including cardiovascular diseases and the number of studies on miRNAs in relation to cardiovascular pathologies has exponentially increased [5,6,7]. It is known that miRNAs are present in circulating blood in exosomes [8] and microparticles [9]. It has also been reported that miRNAs are bound to other RNA binding proteins, including nucleophosmin 1 (NPM1), Argonaute2 (Ago2), or by HDL instead of vesicles and are stable in blood [10,11,12]. The levels of specific, circulating miRNAs have been shown to be associated with various pathological conditions [13,14], such as heart failure [15] and acute coronary syndrome (ACS) [16,17,18,19,20]. Moreover, accumulating evidence suggests that circulating miRNAs exist not only in serum but also in other body fluids [21]. From these results, we hypothesized that miRNAs in pericardial fluid (PF) also may reflect the condition of the heart.

It has been reported that miR-423-5p levels are higher in patients with HF compared with healthy controls [22,23]. The hearts of patients who need to undergo open heart surgery possibly have repeated ischemic or hemodynamic stresses, which are associated with HF. Therefore, in this report, we measured the levels of miR-423-5p in serum and PF from patients treated with a coronary artery bypass graft (CABG) or aortic valve replacement due to aortic stenosis (AS). We also compared these levels with those of muscle-enriched miR-133a and vascular-enriched miR-126 and miR-92a in the same samples to address the source of miR-423-5p.

## Materials and Methods

### Study Population

This study was approved by the Institutional Review Board of Kyoto University Graduate School and Faculty of Medicine and Kitano Hospital. Written informed consent was given by all patients or their families in accordance with the Declaration of Helsinki. Diagnosis was based on the final diagnosis at discharge, which relied on the treating physician’s diagnosis.

### Blood and Cardiac Effusion Sampling from Patients

Serum (S) and PF from patients undergoing coronary artery bypass graft (CABG) due to stable angina pectoris (sAP) and unstable AP (uAP) and aortic valve replacement due to AS were analyzed for the detection of circulating miRNAs. These samples were designated S-sAP, S-uAP, S-AS, PF-sAP, PF-uAP, and PF-AS. Venous blood obtained on admission was put into a commonly used test tube containing polyolefin resin for serum separation. The tube was centrifuged at 12,000 × g for 10 min. PF samples were collected during operation. The serum and PF was aliquoted and stored at -80°C until assayed.

### Quantification of Circulationg miRNAs

As described in detail previously [20], we quantified circulating miRNAs. Total RNA was extracted from 350μL of serum and PF, using TRIzol LS reagent (Invitrogen, Carlsbad, USA). microRNAs were quantified using quantitative reverse-transcriptase-polymerase chain reaction (qRT-PCR) TaqMan MicroRNA Assays (Applied Biosysetms, Foster city, USA) and a 7900HT Fast Real-Time PCR System (Applied Biosystems) in accordance with the manufacturer’s instructions. Total RNA (100ng) was used to synthesize miR-133a-specific cDNA, and 10 ng of total RNA was used to synthesize miR-423-5p-, miR-126-, and miR-92a-specific cDNAs using the TaqMan microRNA assay. To evaluate miRNA expression levels, PCR was carried out in duplicate. Each miRNA level was normalized using exogenous cel-miR-39 and quantified by the 2^-ΔCt^ method. When the Ct value of cel-miR-39 could not be determined or was more than 31, the samples were thought to be degradated and were excluded. If the Ct value of the target miRNA could not be determined, the expression level was regarded as 0.

### Evaluation of the Stability of Double Strand RNAs

RNA oligonucleotides were dissolved at 5μM in RNAse free water and annealed. RNaseA (Roche, Basel, Switzerland) was added to the annealed RNA oligonucleotides at a concentration of 100ug/ml. Total RNA was extracted from RNA solutions before and 5 and 20minutes after the addition of RNaseA using TRIzol LS reagent (Invitrogen), Each miRNA level was normalized with exogenous cel-miR-39 and quantified by the 2^-ΔΔCt^ method. Total RNA (100 ng) was used to synthesize miR-423-5p-specific cDNA using TaqMan microRNA assay. For the measurement of each miRNA, 7900HT Fast Real-Time PCR System was used, as described for the quantification of circulating miRNA. RNA oligonucleotides sequence used in this assay were as follows.

hsa-miR-423-5p: UGAGGGGCAGAGAGCGAGACUUU,

hsa-miR-3184-3p: AAAGUCUCGCUCUCUGCCCCUCA,

and hsa-miR-133a-3p: UUUGGUCCCCUUCAACCAGCUG.

### Purification of Neonatal Mouse Cardiac Myocytes and Fibroblasts

We purified neonatal mouse cardiomyocytes and cardiac fibroblasts in according with previous reports [24,25]. Mice were maintained on a 12-h light/dark cycle, fed a normal laboratory diet ad libitum, sacrificed by decapitation. Mouse ventricles were isolated from 1-day-old C57BL/6 mice and dispersed in digestion buffer containing 1.3 mg/mL pancreatin (P3292; Sigma, St.Louis, USA) and 0.45 mg/mL collagenase type II (17101–015; Gibco^®^, Invitrogen Carlsbad, USA). Collected cells were washed in a 3:1 mixture of Dulbecco’s modified Eagle’s medium (DMEM) and medium 199 (Gibco^®^) supplemented with 10% horse serum (Gibco^®^), 10% fetal bovine serum (FBS; Sigma, 172012), 100 units/mL penicillin (Gibco^®^), 100 μg/mL streptomycin (Gibco^®^), and 292 μg/mL L-glutamine (Gibco^®^). The cells were centrifuged at 280 ×g for 5 min, mixed in serum-free DMEM and stained for fluorescence-activated cell sorting (FACS) analyses. MitoTracker^®^ Green FM (M7514; Invitrogen, Carlsbad, USA) solution was utilized to identify cardiac myocytes, and anti-mouse Thy-1.2 antibody conjugated with allophycocyanin (APC) (17-0902-81; eBioscience, San Diego, USA) was used to stain cardiac fibroblasts. FACS system (BD FACSAria^™^ II; Becton Dickinson, Franklin Lakes, USA) was used to detect MitoTracker Green and APC. This investigation conformed to the Guide for the Care and Use of Laboratory Animals published by the US National Institutes of Health (NIH Publication No. 85–23, revised 1996). All animal care, experiments, and methods were approved by the Animal Care and Use Committees of Kyoto University Graduate School of Medicine (MedKyo15167).

### Statistical Analysis

Data are presented as mean ± SD for patient characteristics, as median and interquartile ranges for human samples and as mean ± SEM in in vitro experiments, unless otherwise described. Patient characteristics were assessed using one-way ANOVA and chi-square test. For statistical comparisons, Wilcoxon test (two paired groups), Spearman test (two groups), or Kruskal-Walllis test (three groups) with Dunn’s multiple comparisons test were used in for human samples. For statistical comparisons for *in vitro* experiments, Student’s t test (two unpaired group) or ANOVA (three or more groups) with Turkey’s post hoc test were used. A probability value <0.05 was considered to indicate statistical significance. When the Ct value of cel-miR-39 could not be determined or was more than 31, the samples were thought to be degraded and were excluded. Statistical analyses were performed using GraphPad Prism 6 (GraphPad Software, Inc., San Diego, USA) or JMP Pro version 11 statistical packages (SAS Institue Inc., Cary, USA).

## Results

### miR-423-5p is Enriched in the PF and Is Elevated in S-uAP Compared with S-sAP and S-AS

**Fig 1:**
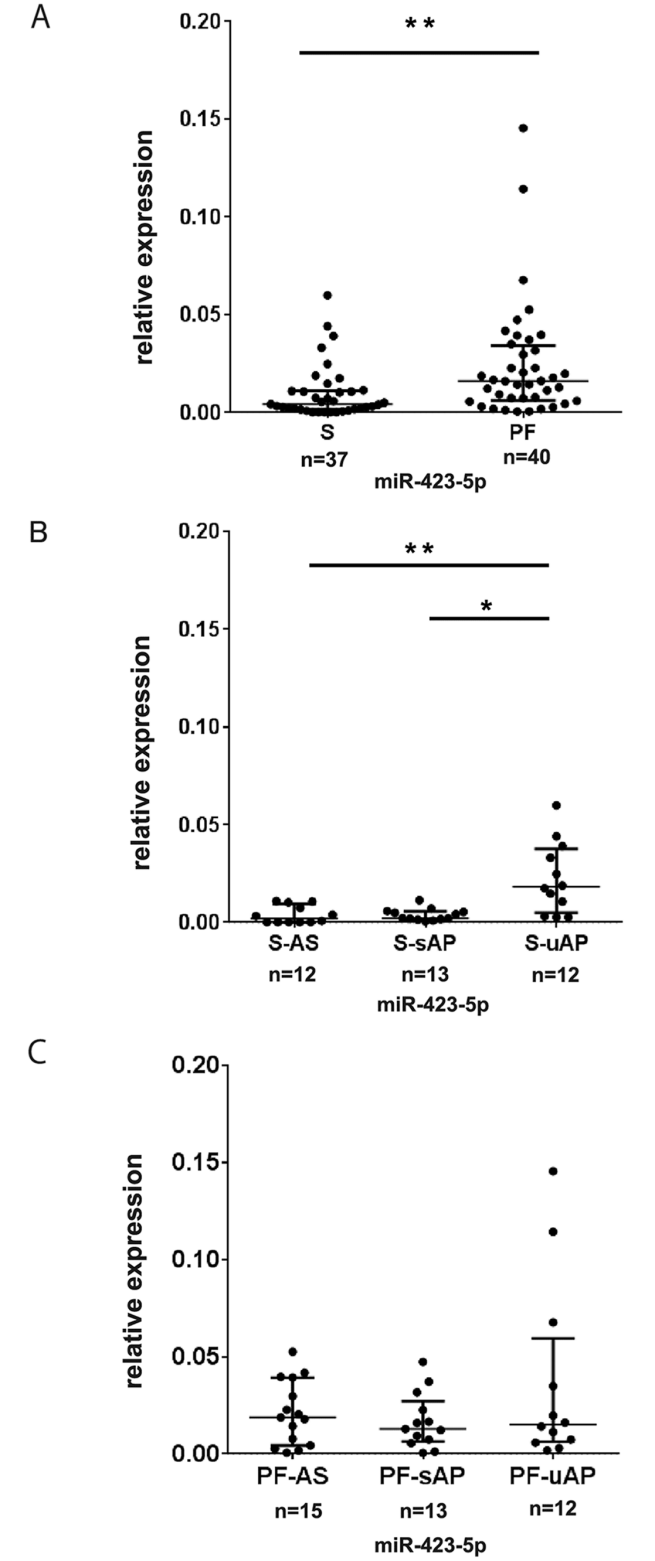
Serum miR-423-5p levels are elavated in the patients with unstable angina pectoris (uAP) compared with stable angina pectoris (sAP) and aortic stenosis (AS). (A) Expression levels of miR-423-5p in the pericardial fluid (PF) and serum. (B) Expression levels of miR-423-5p in serum of patients with AS, sAP and uAP. miR-423-5p levels were using exogenous cel-miR-39. Data are presented as mean ± SD. *P<0.*05*. **P<0.*01*. (C) Expression levels of miR-423-5p in the PF of patients with AS, sAP and uAP. miR-423-5p levels were normalized using exogenous cel-miR-39. Data are presented as median and interquartile range. *P<0.*05*.

To clarify the expression levels of miRNAs in PF in relation with those in the serum in patients undergoing cardiac surgery, serum and PF from patients undergoing CABG due to sAP (n = 16) and uAP (n = 13) and aortic valve replacement due to AS (n = 13) were analyzed. Accordingly, we designated these samples S-sAP, S-uAP, S-AS, PF-sAP, PF-uAP, and PF-AS. Quantitative real-time PCR methodologies have been widely applied in miRNA research, especially for assessing low levels of serum miRNAs. To date, the most widely used and successful approach in terms of specificity and sensitivity is a two-step approach using looped miRNA-specific reverse transcription primers and TaqMan probes [26]. Therefore, we applied this system to determine the levels of miRNAs in our patients. Baseline characteristics of the patients according to their disease condition are summarized in Table 1. The aspartate aminotransferase and alanine aminotransferase levels of the patients with uAP were significantly higher than in those with sAP and AS. The proportion of patients reporting regular alcohol intake was also significantly elevated in patients with uAP compared with the other groups. We first measured the levels of miR-423-5p, which was recognized previously as a biomarker for heart failure. We found that miR-423-5p levels were significantly higher in PF compared with serum (Fig 1A). Although there was no difference in the levels of miR-423-5p among PF-AS, PF-sAP, and PF-uAP, (Fig 1B), miR-423-5p levels were significantly higher in S-uAP than those in S-AS and S- sAP (Fig 1C).

**Table 1:** Baseline Characteristics of Patients with sAP, uAP, and AS.

	Stable AP (N = 16)	Unstable AP (N = 13)	AS (N = 16)	p value
Age (yr), N = 45	71.4±10.0	71.6±3.6	71.8±13.1	n.s
Gender (male/female), N = 45	10/6	10/3	9/7	n.s
Height (cm), N = 45	158.9±8.6	162.5±8.0	159.4±8.2	n.s
Weight (kg), N-45	61.3±12.6	65.5±12.9	61.2±11.5	n.s
LVDd (mm), N = 42	48.9±8.4	52.0±6.1	47.7±6.5	n.s
LVDs (mm), N = 42	35.2±10.2	38.2±8.5	33.1±6.2	n.s
LVEF (%), N = 42	54.9±11.8	50.2±15.8	58.9±7.7	n.s
Diabetes mellitus (%), N = 45	50.0	46.2	43.8	n.s
Hypertension (%), N = 45	87.5	92.3	68.8	n.s
Dyslipidemia (%), N = 45	81.3	69.2	62.5	n.s
Active Smoker (%), N = 43	6.7	16.7	6.3	n.s
Alcohol (%), N = 41	46.7	80.0	25.0	0.02
ACEI or ARB (%), N = 45	62.5	69.2	56.3	n.s
β-blocker (%), N = 45	25.0	30.8	31.3	n.s
Oral hypoglycemic drug (%), N = 45	37.5	23.1	31.3	n.s
Insulin (%), N = 45	18.8	15.4	12.5	n.s
Statin or probucol (%), N = 45	56.3	53.9	43.8	n.s
AST (IU/L), N = 42	21.6±7.2	32.6±14.8	18.9±6.4	<0.01
ALT (IU/L), N = 42	17.4±10.0	28.8±17.5	13.3±7.2	<0.01
LDL-cho (mg/dl), N = 42	110.6±33.9	113.4±35.0	104.0±38.1	n.s
CK (mg/dl), N = 44	73.7±34.9	160.0±165.1	111.8±103.8	n.s
Cr (mg/dl), N = 42	1.9±1.7	1.5±2.2	3.4±3.7	n.s
eGFR (ml/min/1.73m^2^), N = 42	40.0±24.3	57.8±20.6	44.1±33.0	n.s
Hb (g/dl), N = 44	12.4±2.0	12.7±2.1	11.6±1.7	n.s
log_2_BNP (pg/ml), N = 42	6.4±2.0	7.3±2.2	7.8±2.7	n.s
BS (mg/dl), N = 44	120.4±47.7	140.2±49.2	146.0±61.7	n.s
HbA1c (%), N = 41	5.9±1.2	6.4±1.9	5.9±1.9	n.s

sAP, stable angina pectoris; uAP, unstable angina pectoris; AS, aortic stenosis; LVDd, left ventricular end-diastolic dimension; LVDs, left ventricular end-systolic dimension; LVEF, left ventricular ejection fraction; ACEI, angiotensin-converting enzyme inhibitor; ARB, angiotensin receptor blocker; AST, aspartate aminotransferase; ALT, alanine aminotransferase; LDL-cho, low density lipoprotein; CK, creatine phosphokinase; eGFR, estimated glomerular filtration rate; Hb, hemoglobin; BNP brain natriuretic peptide; BS, blood sugar; HbA1c, and hemoglobin A1c. All values are expressed as mean ± SD.

### Muscle-Enriched and Vascular-Enriched miRNAs Are Not Elevated in PF

**Fig 2:**
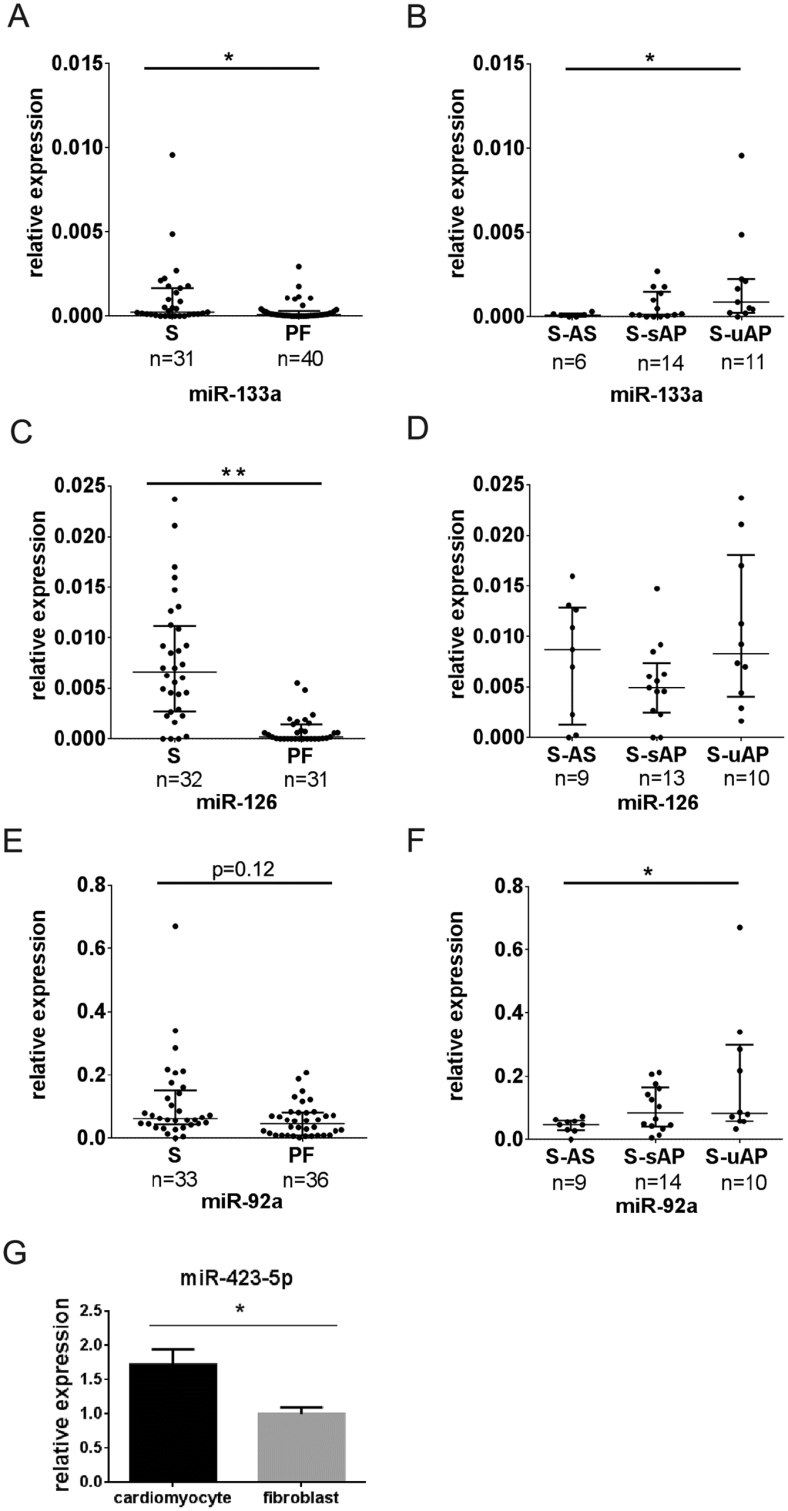
Muscle-enriched and vascular-enriched miRNAs are not elevated in PF compared with serum. (A) Expression levels of miR-133a in serum and PF, (B) Expression levels of miR-133a in the serum of patients with aortic stenosis (AS), stable angina pectoris (sAP) and unstable angina pectoris (uAP), (C) Expression levels of miR-92a in serum and PF, (D) Expression levels of miR-92a in serum of patients with AS, sAP and uAP, (E) Expression levels of miR-126 in serum and PF, (F) Expression levels of miR-126 in the serum of patients with AS, sAP and uAP. Each miRNA level was normalized using exogenous cel-miR-39. Data are presented as median and interquartile range. *P<0.*05*. **P<0.*01*. (G) Expression levels of miR-423-5p in cardiomyocytes and fibroblasts from neonatal mice hearts, n = 4. miR-423-5p was normalized using U6. Data are presented as mean ± SEM. * p<0.*05*

In order to clarify the source of miR-423-5p, we also measured the levels of muscle-enriched miR-133a and vascular-enriched miR-126 and miR-92a in the same samples. The levels of miR-126 and miR-133a were significantly elevated in the serum compared with PF, and miR-92a showed a similar tendency (P = 0.12) (Fig 2A, 2C and 2E). miR-133a and miR-92a levels were significantly elevated in S-uAP compared with S-AS (Fig 2B and 2F). There was no difference in the levels of miR-126 among PF-AS, PF-sAP, and PF-uAP, (Fig 2D), and there was no difference in miR-133a, miR-92a, and miR-126 levels among PF-uAP, PF-sAP and PF-AS (S1 Fig). Elevation of miR-133a in S-uAP was consistent with our previous results that the serum levels of miR-133 were elevated in patients with myocardial injury [20]. We then sought to determine which cells express miR-423-5p in the heart. Cardiomyocytes and cardiac fibroblasts were isolated by FACS from neonatal mice hearts [24,25]. The expression levels of miR-423-5p were significantly higher in cardiomyocytes than in fibroblasts (Fig 2G).

### miR-423-5p and miR-3184 Are Encoded in the Same Intron in the Opposite Direction

**Fig 3:**
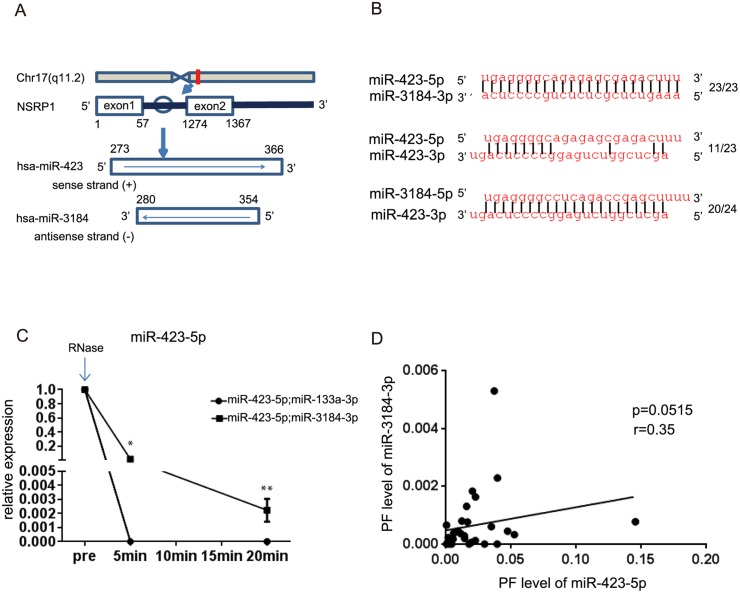
miR-423-5p and miR-3184-3p are complementary to each other. (A) Localization of nuclear speckle splicing regulatory protein 1 (NSRP1) on chromosome 17q11.2. MiR-423-5p is located in intron1 of NSRP1. MiR-423-5p and miR-3184-3p are encoded in opposite direction in the same region. (B) Sequence alignment between miR-423-5p and miR-3184-3p, miR-423-5p and miR-423-3p, and miR-3184-5p and miR-423-5p (C) miR-423-5p:miR-3184-3p and miR-423-5p:miR-133a were treated with 100μg/ml RNase A, and the expression level of miR-423-5p was measured at the indicated time points (n = 3). Data are presented as mean ± SEM. *p<0.*05*, **p<0.*01* (D) The correlation between the expression levels of miR-423-5p and miR-3184-3p in PF (n = 31). Each miRNAs was normalized using exogenous cel-miR-39.

miR-423-5p is located within the intron of nuclear speckle splicing regulatory protein 1 (*NSRP1*). There is also another miRNA, miR-3184, encoded in the opposite direction at the same region (Fig 3A). The complementarity between miR-423-5p are miR-3184-3p is complete, whereas that between miR-3184-5p and miR-423-3p is lower. The complementarity between miR-423-5p and -3p is almost half of that of miR-423-5p and miR-3184-3p (Fig 3B). It was reported that complementary miRNA pairs form miRNA:miRNA duplexes [27]. We hypothesized that miR-423-5p and miR-3184-3p may form miR-423-5p:miR-3184-3p RNA duplexes and analyzed the stability of miR-423-5p:miR-3184-3p duplexes. We annealed miR-423-5p with miR-3184-3p or miR-133a-3p and compared the levels of miR-423-5p using RNaseA treatment for different time periods (Fig 3C). The levels of miR-423-5p were still high at 5 and 20 minutes after the addition of RNase A when an RNA duplex of miR-423-5p:miR-3184-3p formed, which was more stable than that of miR-423-5p:miR-133a-3p. Moreover, the levels of miR-423-5p in PF had a tendency to correlate to the levels of miR-3184-3p (Fig 3D, r = 0.35, p = 0.0515). These results suggested that miR-3184-3p may help to stabilize miR-423-5p in the PF.

## Discussion

In this report, we analyzed miRNA levels in PF in association with those in the serum. miR-423-5p levels were significantly higher in PF than serum. Although there was no difference in miR-423-5p levels among the PF-AS, PF-sAP, and PF-uAP, its levels were significantly elevated in S-uAP compared with those in S-AS and S-sAP. In order to clarify the source of miR-423-5p, we measured the levels of muscle-enriched miR-133a and vascular-enriched miR-126 and miR-92a in the same samples. However, their expression patterns were distinct from miRNA-423-5p. We noticed that miR-423-5p and miR-3184-3p are located within the same region of *NSRP1* intron in the opposite direction, and RNA duplexes may be formed by these miRNAs.

Although the biological function of miR-423-5p is still unknown, we found that miR-423-5p levels were significantly higher in S-uAP than those in S-sAP and S-AS. This result indicated that circulating miR-423-5p may reflect the disease status of cardiac ischemia. It has been reported that miR-423-5p levels are higher in patients with HF compared with healthy controls and correlated with NT-proBNP (BNP) and EF [22]. Other reports also revealed that four circulating miRNAs (miR-423-5p, miR-320a, miR-22 and miR-92b) were significantly increased in patients with systolic HF, and a score based on the levels of these four miRs correlated with clinical parameters in patients with systolic HF [23]. However, there was no correlation between serum or PF levels of miR-423-5p and BNP in our samples (S2 Fig). This may be because our samples were obtained from patients before operations and most of the patients were already controlled by medications. As a result, BNP levels in our samples were not as high as those observed in acute decompensated HF.

Some miRNAs have been shown to exhibit tissue-specific expression, such as miR-133a, miR-126, or miR-92a. On the other hand, miR-423-5p was shown to be ubiquitously expressed in diverse tissues in porcine [28]. Although the origin of PF is unclear, many substances in PF are reported to be carried from coronary sinus through the capillary network [29,30]. miR-423-5p was reported to show significant differences in the transcoronary gradient in HF patients compared with controls. This result suggested that miR-423-5p is produced and/or released by the myocardium in HF patients [31]. Although miR-423-5p is enriched in cardiomyocytes compared with fibroblasts in neonatal mouse heart, the reason why they are elevated in the samples of patients with uAP is still unknown.

In our study, miR-423-5p was detected with high abundance in PF compared with serum. We speculate that miR-423-5p may form a duplex with miR-3184-3p, which protects it from degradation. Currently, it is not possible to identify the existence of duplex RNA *in vivo*. Advanced techniques in the future may enable us to understand the function of the unique localization of miR-423 and miR-3184.

Circulating miRNAs have been reported to be useful as biomarkers of numerous diseases. Weber et al. investigated the distribution of miRNAs in 12 human body fluids such as saliva, urine, and pleural effusion. The composition of miRNAs varied among those samples [32]. Among them, it is reported that several miRNAs in urine samples had potential diagnostic biomarkers of prostate cancer. [33,34]. Kuosmanen et al. made the first report of the profile of miRNAs in PF of patients with HF [35]. They reported that miR-16 and miR-21 were detected at high levels from the PF samples. As shown in S3 Fig, miR-16 and miR-21 were detected at high levels. Although it is impossible to compare different miRNAs in absolute values by using the TaqMan microRNA assay, miR-16 and miR-21 seem to be present at high levels in PF. They could not detect any miRNAs that reflected the clinical features of their patients. However, Fujita et al. showed that angiogenic growth factors in PF were significantly higher in patients with ischemic heart diseases than in those with non-ischemic heart diseases [36]. They also showed that BNP levels in PF were associated with left ventricular dysfunction [37]. Therefore, it was hypothesized that there may be functional miRNAs in PF, and some of them may be utilized to reflect clinical status. Although we were not able to detect the disease-specific miRNAs in PF so far, in depth analyses of miRNAs by miRNA microarrays or RNA sequence may allow us to find miRNAs that are useful to detect the patients’ heart condition in the future.

## Supporting Information

S1 Fig Levels of Muscle-enriched and vascular-enriched miRNAs in pericardial fluid (PF) do not show any difference among patients. (DOCX)

S2 Fig Levels of miR-423-5p in serum or PF are not correlated with log_2_ BNP. (DOCX)

S3 Fig Levels of miR-16 and miR-21 in PF. (DOCX)
