# Supplementary material for: Expression Patterns of miRNA-423-5p in the Serum and Pericardial Fluid in Patients Undergoing Cardiac Surgery
Source: PLoS One. 2015 Nov 12;10(11):e0142904. doi: 10.1371/journal.pone.0142904 (PMC4642962; doi:10.1371/journal.pone.0142904)
Supplement: S1 Fig — (DOCX) [file pone.0142904.s001.docx]

A


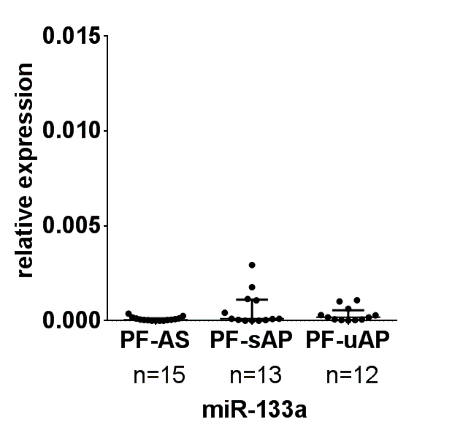


B


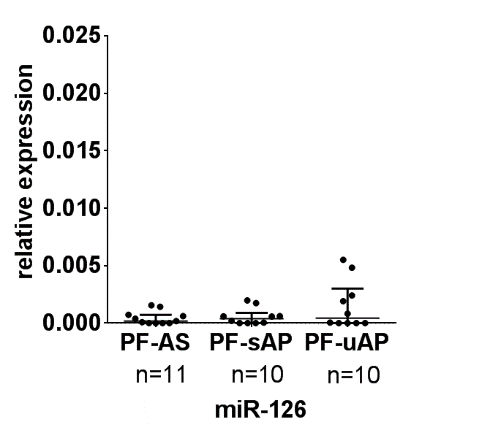


C


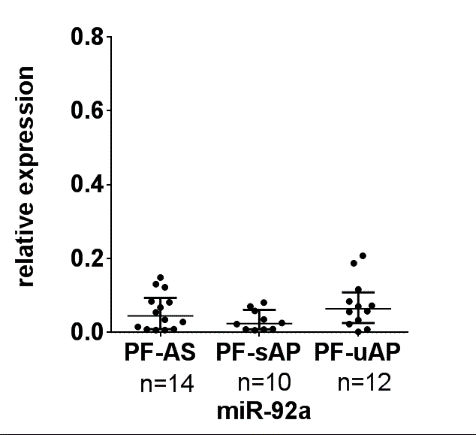


**Supplemental Figure1. Levels of Muscle-enriched and vascular-enriched miRNAs in pericardial fluid (PF) do not show any difference among patients.**

(A) Expression levels of miR-133a in the PF of patients with aortic stenosis (AS), stable angina pectoris (sAP) and unstable angina pectoris (uAP), (B) Expression levels of miR-126 in the PF of patients with AS, sAP and uAP, (C) Expression levels of miR-92a in the PF of patients with AS, sAP and uAP. Each miRNAs was normalized with exogenious cel-miR-39. Data are presented as median and interquartile ranges.
