# Supplementary material for: Expression Patterns of miRNA-423-5p in the Serum and Pericardial Fluid in Patients Undergoing Cardiac Surgery
Source: PLoS One. 2015 Nov 12;10(11):e0142904. doi: 10.1371/journal.pone.0142904 (PMC4642962; doi:10.1371/journal.pone.0142904)
Supplement: S2 Fig — (DOCX) [file pone.0142904.s002.docx]

A

B

**Supplemental Figure 2. Levels of miR-423-5p in serum or PF are not correlated with log_2_ BNP.**

A and B, Spearman correlation between log_2_BNP and miR-423-5p in the serum (A) or in PF (B).
