# Supplementary material for: Expression Patterns of miRNA-423-5p in the Serum and Pericardial Fluid in Patients Undergoing Cardiac Surgery
Source: PLoS One. 2015 Nov 12;10(11):e0142904. doi: 10.1371/journal.pone.0142904 (PMC4642962; doi:10.1371/journal.pone.0142904)
Supplement: S3 Fig — (DOCX) [file pone.0142904.s003.docx]

A


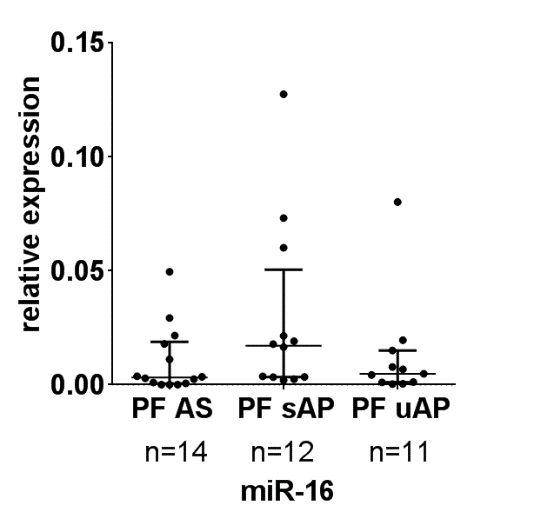


B


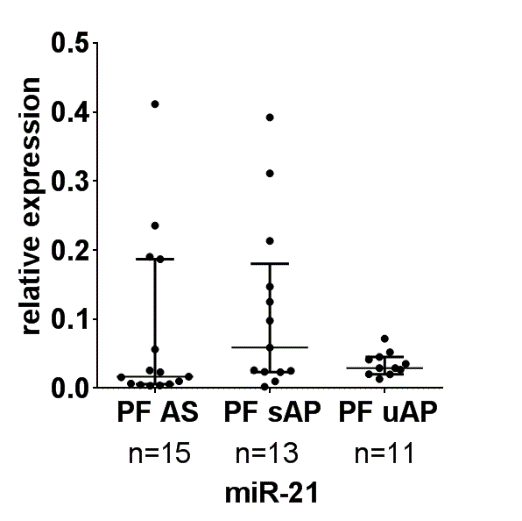


**Supplemental Figure 3. Levels of previously described abundant miRs in pericardial fluid (PF) are present in these samples.**

(A) Expression levels of miR-16 in the PF of patients with aortic stenosis (AS), stable angina pectoris (sAP) and unstable angina pectoris (uAP), (B) Expression levels of miR-21 in the PF of patients with AS, sAP and uAP, Each miRNAs was normalized with exogenious cel-miR-39. Data are presented as median and interquartile ranges.
